# Supplementary material for: Defining relative mutational difficulty to understand cancer formation
Source: Cell Discov. 2020 Jul 21;6:48. doi: 10.1038/s41421-020-0177-8 (PMC7371891; doi:10.1038/s41421-020-0177-8)
Supplement: Supplementary file 1 — Supplementary Information [file 41421_2020_177_MOESM1_ESM.pdf]

Supplementary information for

Figure S1 Rationale for reverse calculation of relative mutational difficulty in human cancer.

Figure S2 Composition of the 26,154 cancer samples with genomic sequencing data in COSMIC.

Figure S3 Potential impacts of highly-selected cancer mutations on calculation of relative mutational difficulty.

Figure S4 The influence of +2 and -2 nucleotides on relative mutational difficulty.

Figure S5 Cancer type-specific mutational difficulties compared with mutational difficulties calculated from all cancer types in average.

Figure S6 Distribution of revised counts of p53 synonymous mutations.

Figure S7 Saos-2 colony formation assay results for various p53 mutations.

Figure S8. The readings of the GFP-based assay that analyze dominant negative effects of p53 constructs.

Figure S9. Transcriptional activities of different p53 mutants.

Figure S10. Additional functionally important amino acid residues and regions in p53.

Figure S11. Potential implications for cancer prevention.

Figure S12. Potential impact of p53 codon changes on tumorigenesis.

Table S1 Calculation of relative mutational difficulty (All cancer types combined).

Table S2 Cancer type-specific mutational difficulty.

Table S3 Original and revised mutation counts of p53 mutations in COSMIC database.

Table S4 List of p53 mutations whose functionalities are annotated differently by revised counts method and FATHMM.

Table S5 List of p53 mutations whose functionalities are annotated differently by revised counts method and PHANTM.

Table S6 List of p53, PTEN and INK4A mutants analyzed in this study.

Table S7 List of primers used for mutagenesis experiments.

Table S8 List of primers used for qPCR analysis.

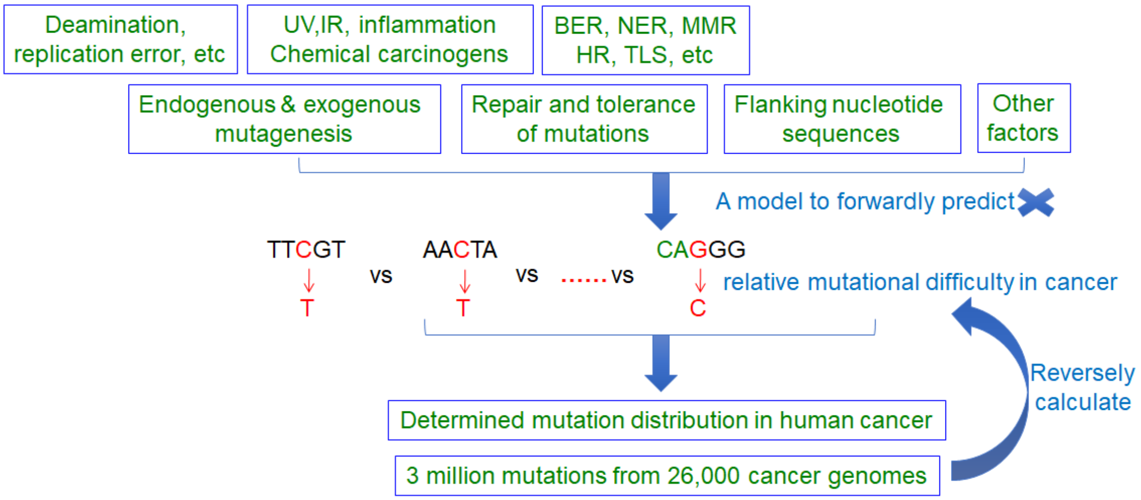

Figure S1. Rationale for reverse calculation of relative mutational difficulty in human cancer.

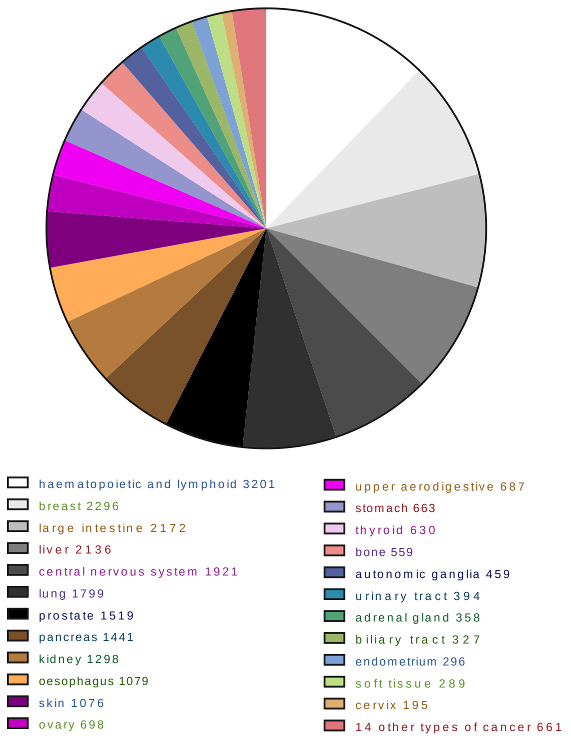

Figure S2. Composition of the 26,154 cancer samples with genomic sequencing data in COSMIC. The numbers following each cancer type indicate how many cancer genomes of the corresponding cancer type are analyzed.

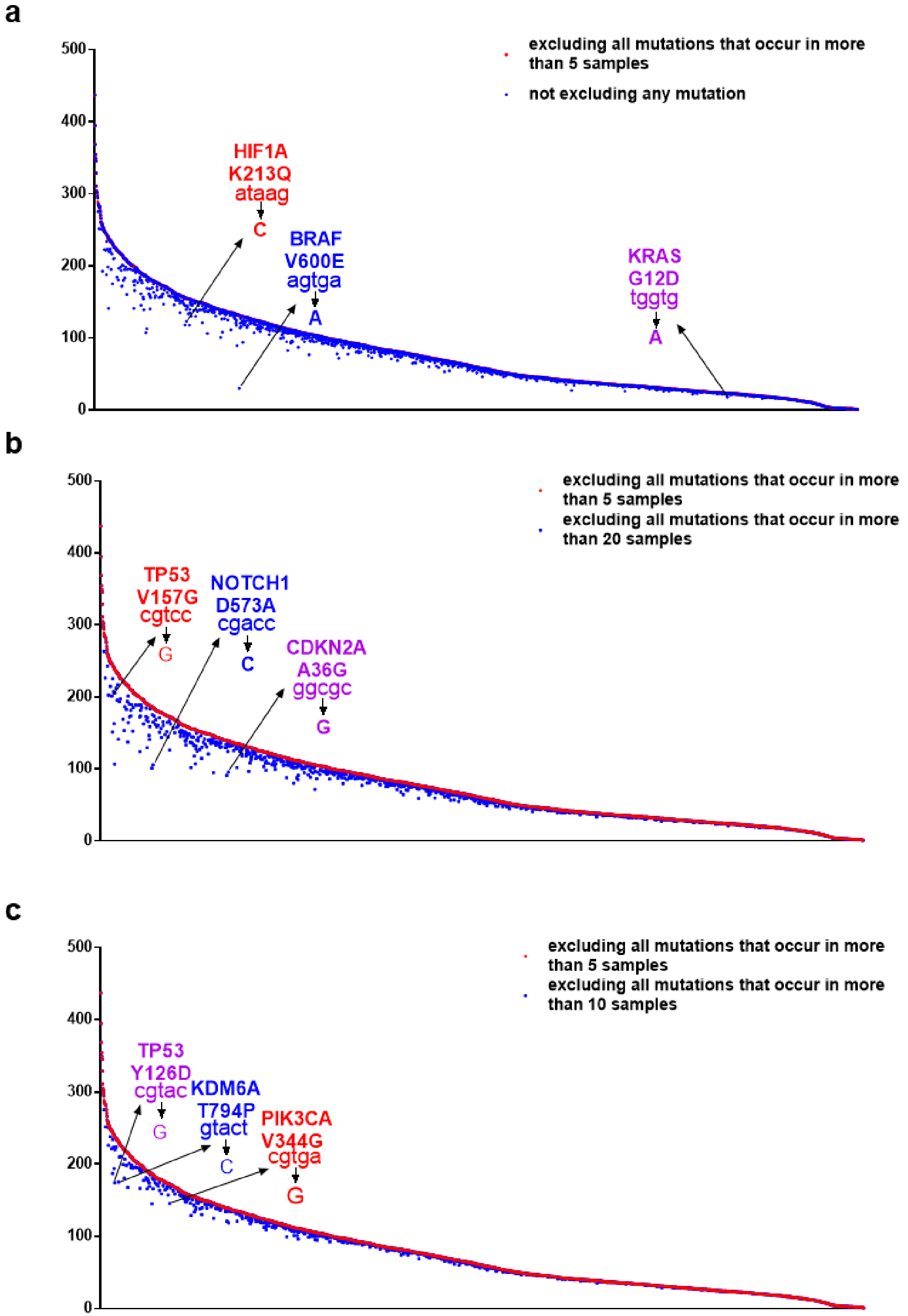

Figure S3. Potential impacts of highly-selected cancer mutations on calculation of relative mutational difficulty. Certain cancer-driving mutations such as BRAF V600E

are highly selectively enriched in cancer. The number of such mutations are significantly increased in the dataset, not because they are easy to generate, but because they are strongly enriched during the tumorigenesis process. Therefore, their presence in the dataset may skew our estimation of the natural mutational tendency for each type of mutation. In our analysis, we excluded cancer mutations that occur in more than 5 cancer samples. The above panels show if no mutations are excluded (a), excluding mutations that occur in more than 20 (b) or 10 (c) samples, what is the impact on calculation of relative mutational difficulty. For example, if no mutations are excluded, the mutational difficulty for T to A substitution on a AGTGA sequence, which underlies the BRAF V600E mutation, will appear significantly lower. Several other similar examples are marked out in the above panels.

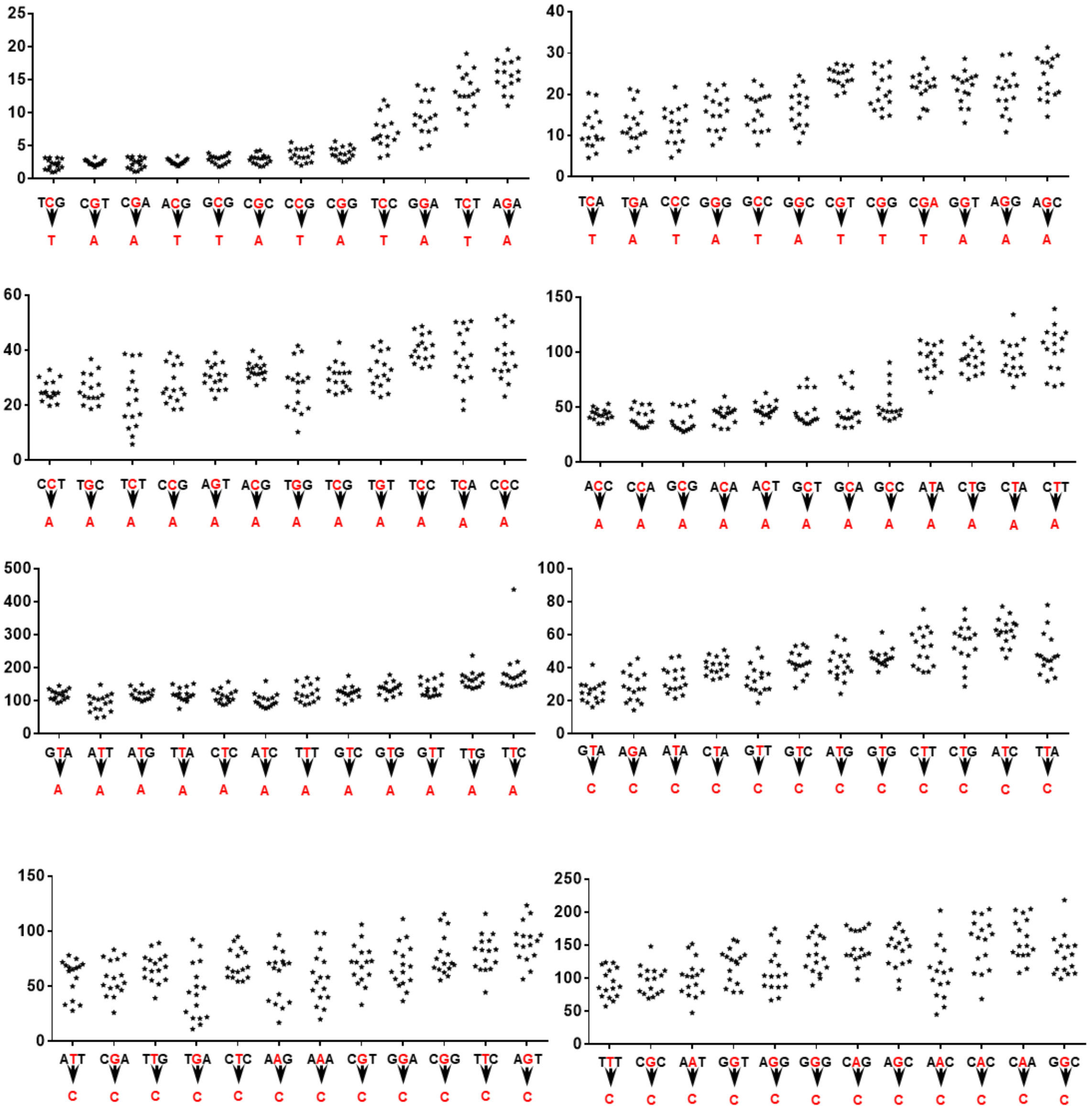

0 5 10 15 20 25
TCG CGT CGA ACG GCG CGC CCG CGG TCC GGA TCT AGA
T A A T T A T A T A T A
0 10 20 30 40
TCA TGA CCC GGG GCC GGC CGT CGG CGA GGT AGG AGC
T A T A T A T T T A A A
0 20 40 60
CCT TGC TCT CCG AGT ACG TGG TCG TGT TCC TCA CCC
A A A A A A A A A A A A
0 50 100 150
ACC CCA GCG ACA ACT GCT GCA GCC ATA CTG CTA CTT
A A A A A A A A A A A A
0 100 200 300 400 500
GTA ATT ATG TTA CTC ATC TTT GTC GTG GTT TTG TTC
A A A A A A A A A A A A
0 20 40 60 80 100
GTA AGA ATA CTA GTT GTC ATG GTG CTT CTG ATC TTA
C C C C C C C C C C C C
0 50 100 150
ATT CGA TTG TGA CTC AAG AAA CGT GGA CGG TTC AGT
C C C C C C C C C C C C
0 50 100 150 200 250
TTT CGC AAT GGT AGG GGG CAG AGC AAC CAC CAA GGC
C C C C C C C C C C C C

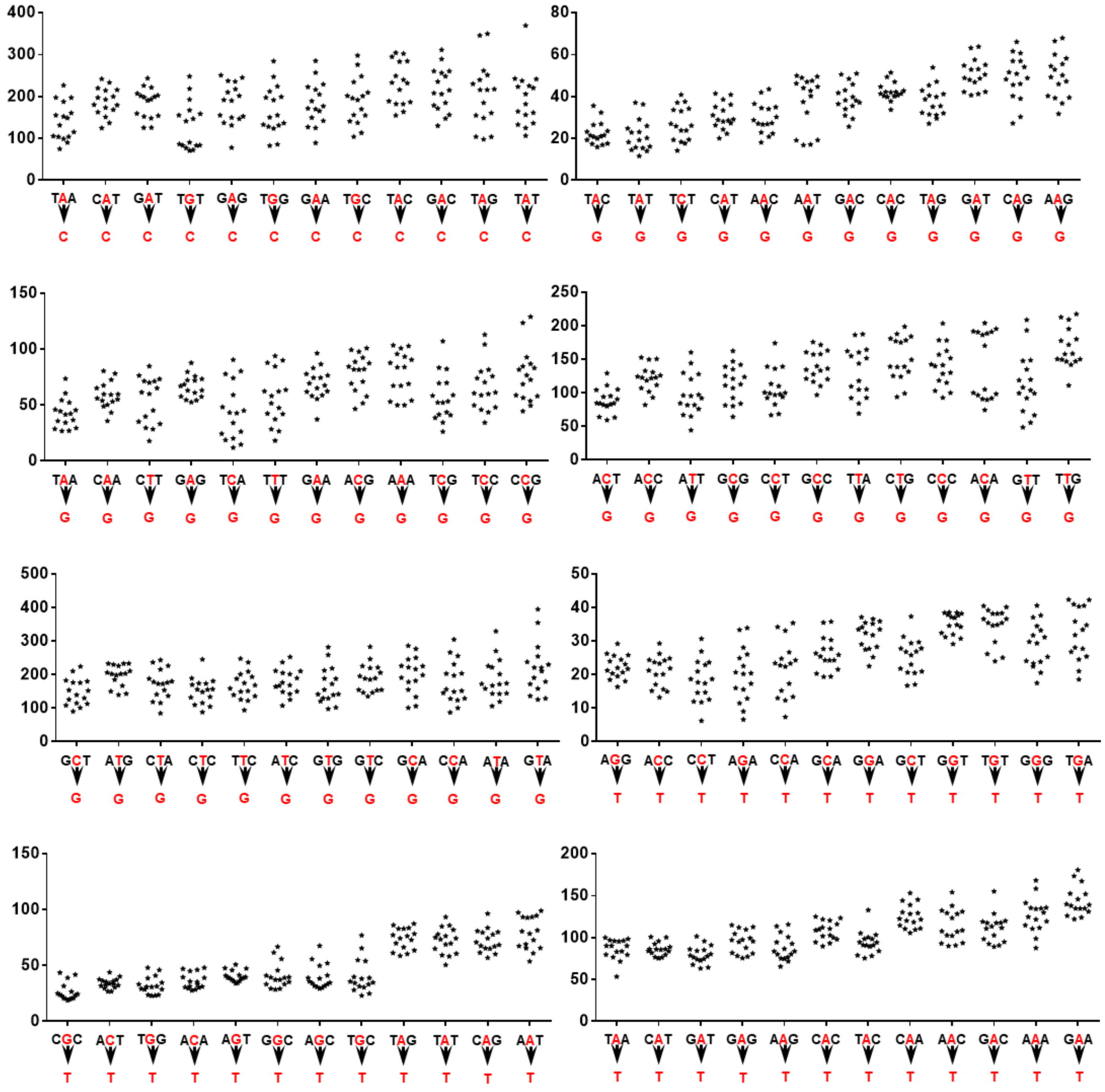

Figure S4. The influence of +2 and -2 nucleotides on relative mutational difficulty. In the above panels, the y-axis represents relative mutational difficulty. For example, in the last column of the lowest right panel, the sixteen * indicate on a NGAGN sequence, how 16 variations of the nucleotide in the +2 and -2 position will impact the relative mutational difficulty for A to T mutation on such NGAGN sequences.

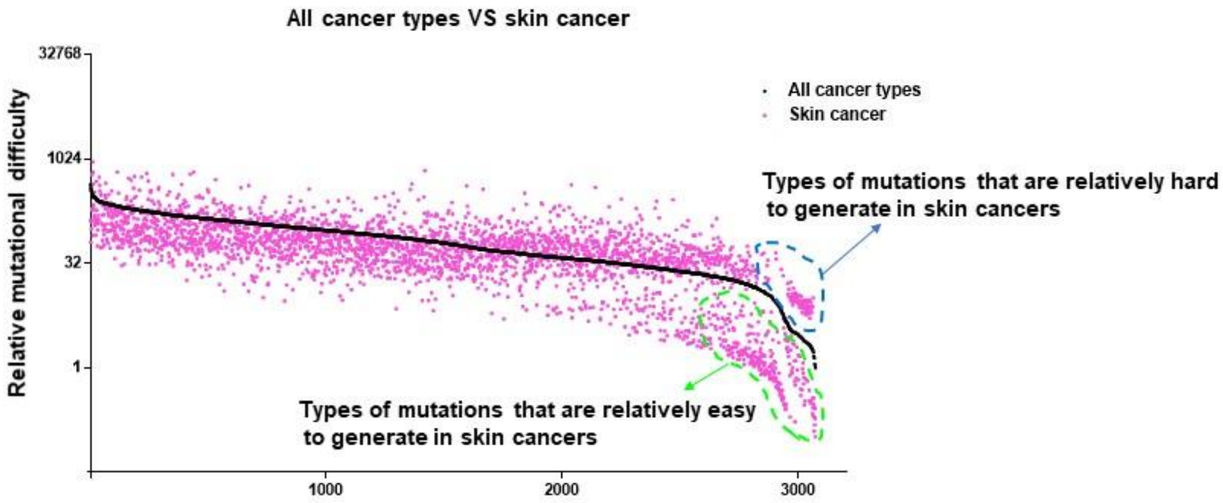
All cancer types VS skin cancer
32768
1024
32
1
Relative mutational difficulty
All cancer types
Skin cancer
Types of mutations that are relatively hard to generate in skin cancers
Types of mutations that are relatively easy to generate in skin cancers
1000
2000
3000

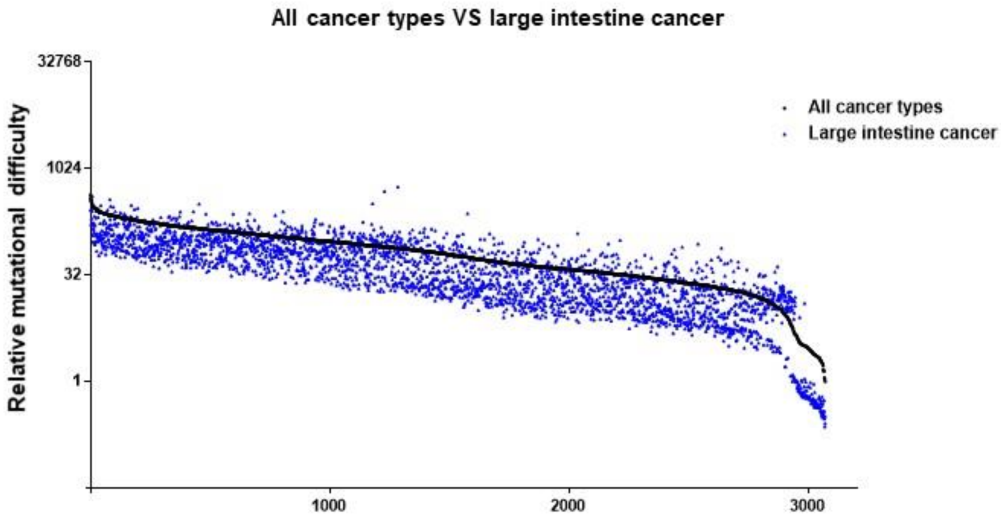
All cancer types VS large intestine cancer
32768
1024
32
1
Relative mutational difficulty
All cancer types
Large intestine cancer
1000
2000
3000

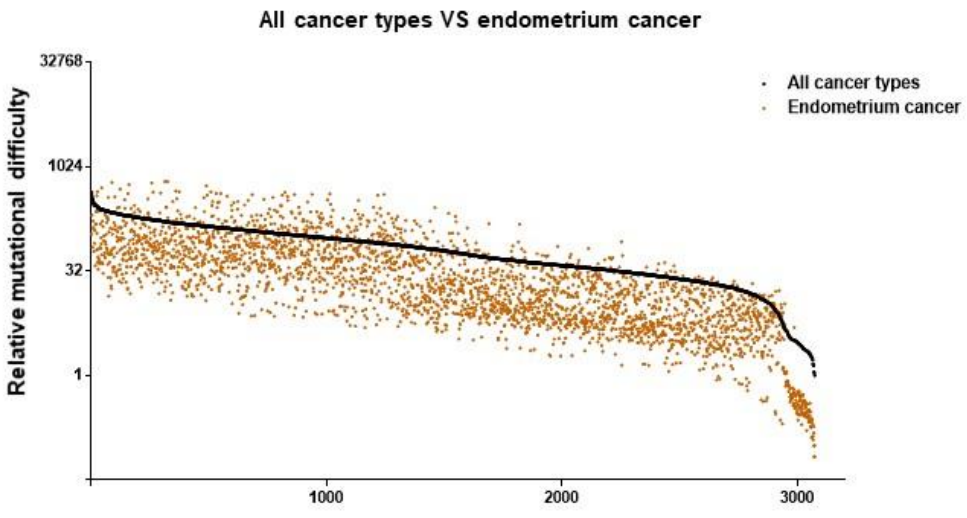
All cancer types VS endometrium cancer
32768
1024
32
1
Relative mutational difficulty
All cancer types
Endometrium cancer
1000
2000
3000

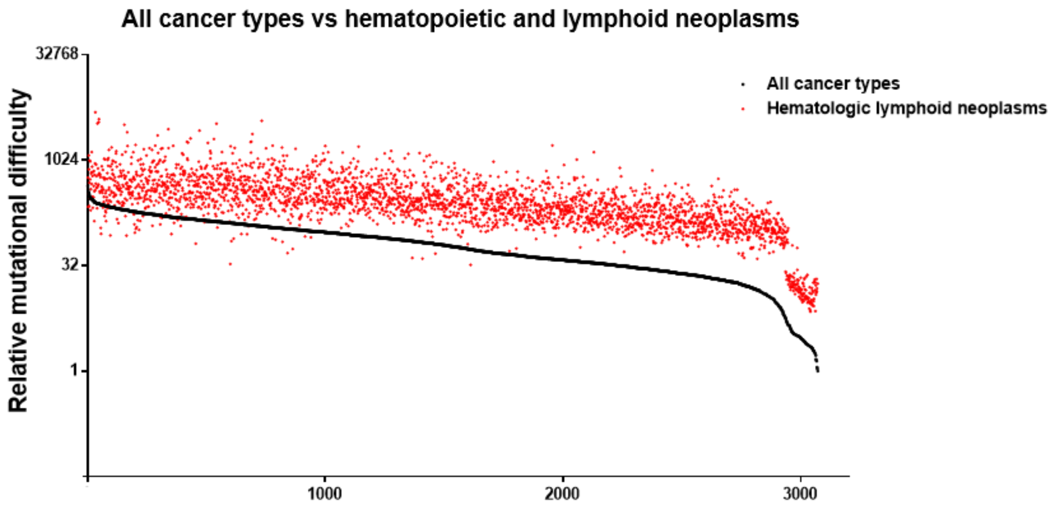
All cancer types vs hematopoietic and lymphoid neoplasms
Relative mutational difficulty
32768
1024
32
1
1000
2000
3000
All cancer types
Hematologic lymphoid neoplasms

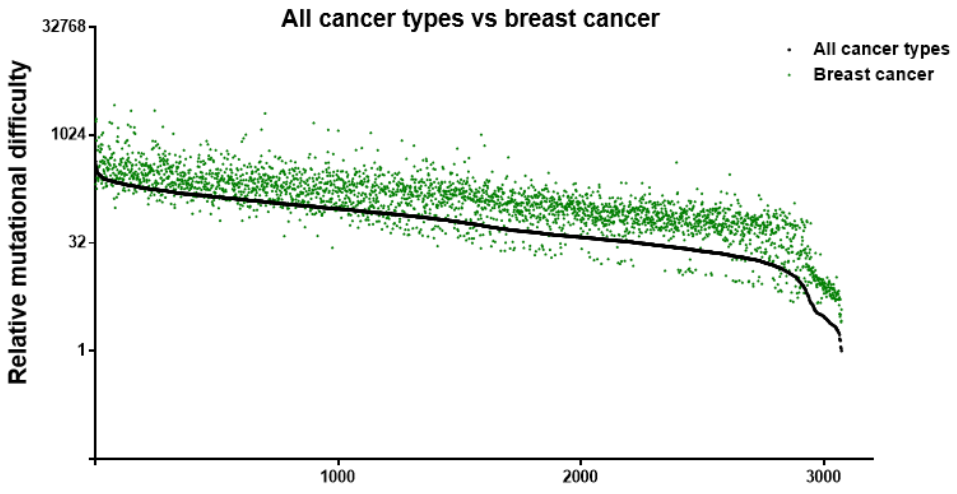
All cancer types vs breast cancer
Relative mutational difficulty
32768
1024
32
1
1000
2000
3000
All cancer types
Breast cancer

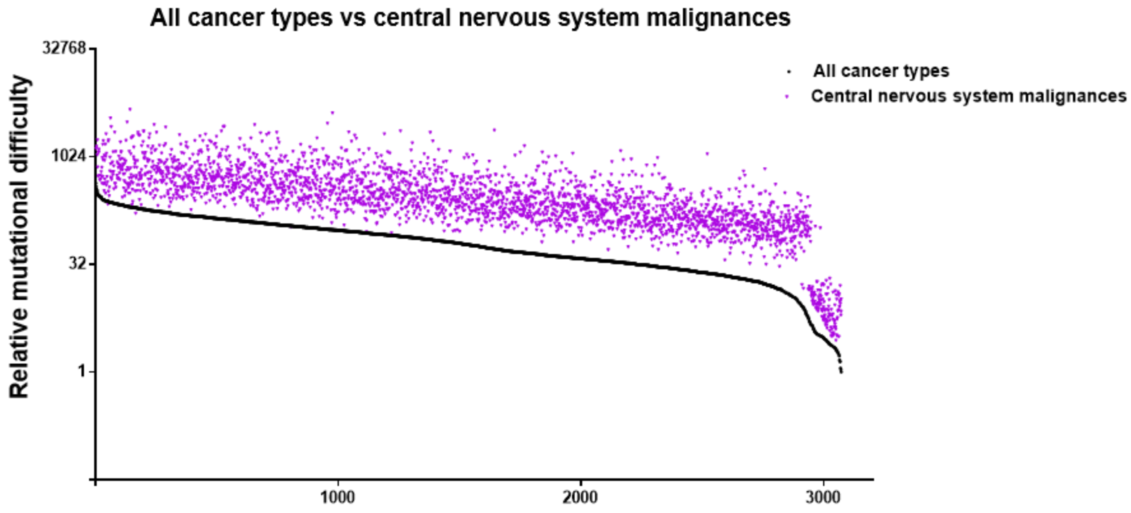
All cancer types vs central nervous system malignances
Relative mutational difficulty
32768
1024
32
1
1000
2000
3000
All cancer types
Central nervous system malignances

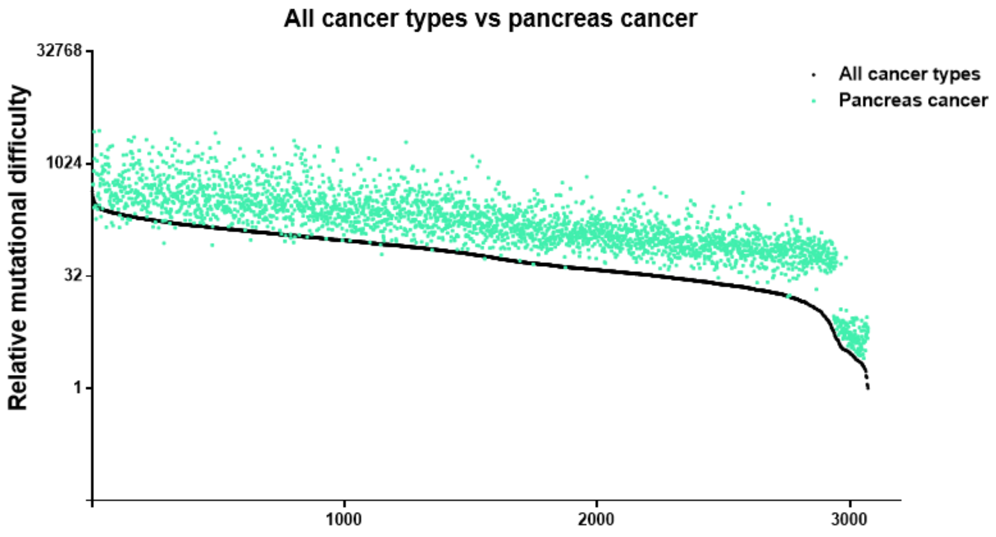

Figure S5. Cancer type-specific mutational difficulties compared with mutational difficulties calculated from all cancer types in average. Black dots indicate relative mutational difficulty scores calculated from 26,154 cancer genomes (all cancer types combined). In the top panel, examples of mutation types that are easier or more difficult to generate in skin cancer are circled out.

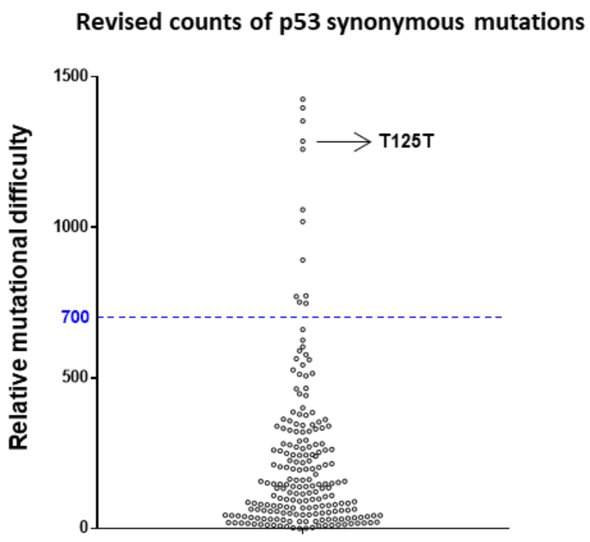

Figure S6. Distribution of revised counts of p53 synonymous mutations. Synonymous p53 mutation data were compiled from the COSMIC database. Shown are revised mutation counts calculated for each type of synonymous p53 mutation. Certain synonymous mutations on p53 are known to be detrimental to the gene. For example, the T125T(c.375 G to A/C/T) mutation disrupts p53 splicing and causes loss of p53 activity[41].

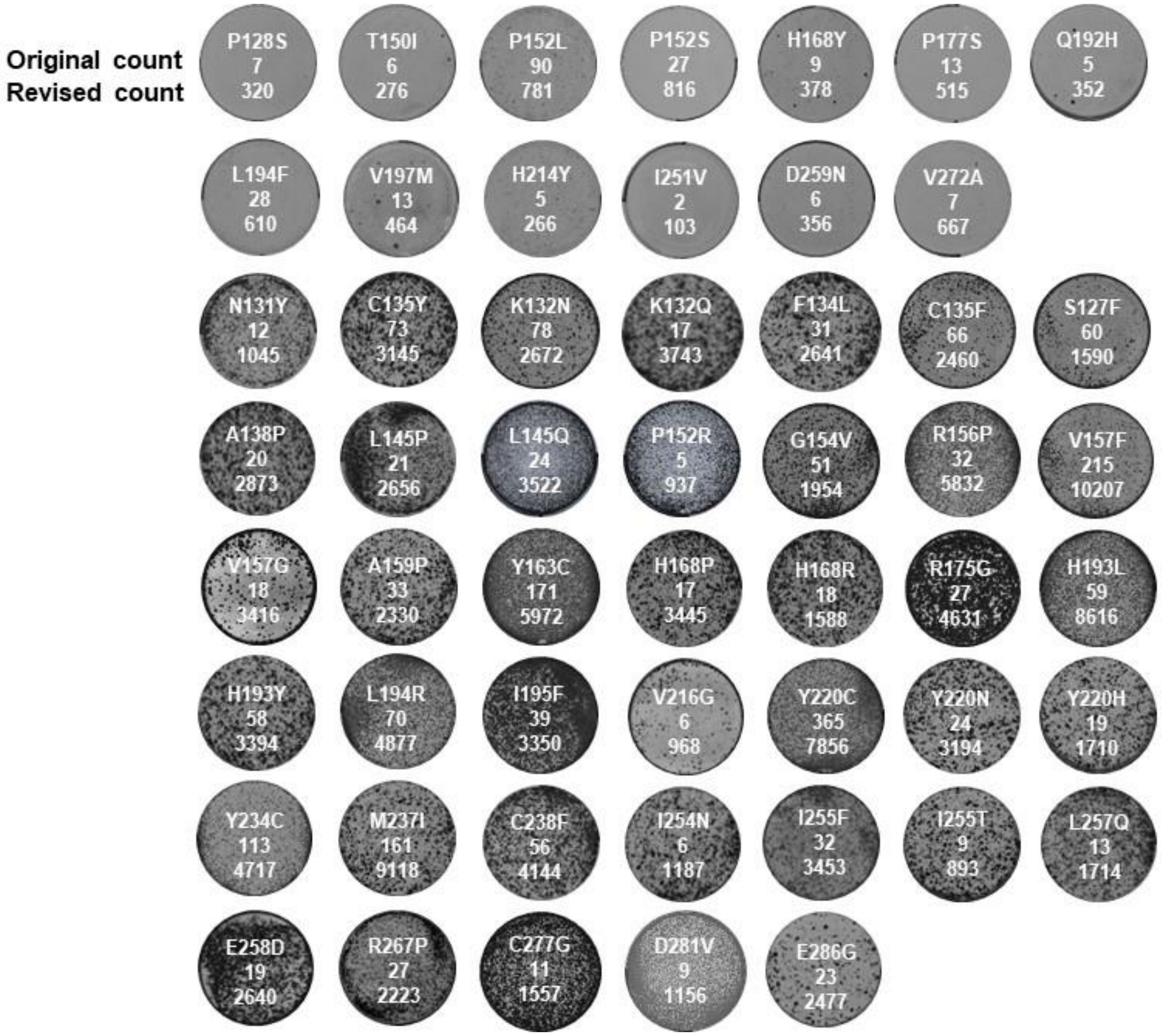

Figure S7. Saos-2 colony formation assay results for various p53 mutations. Shown here are experimental results of all p53 mutations included in this study, in addition to those shown in Figure 2c and 2d. The original counts and revised counts are listed below each mutation.

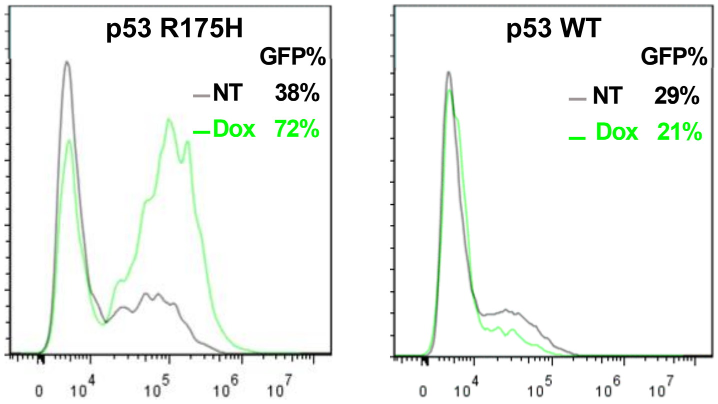

Figure S8. The readings of the GFP-based assay that analyze dominant negative effects of p53 constructs. On the left panel, the co-expression of dominant negative p53 R175H protects cells from DNA damage. Upon treatment with doxorubicin, more GFP positive, p53 R175H expressing cells survived, leading to an increase of percentage of GFP positivity in surviving cells. On the right panel, expression of wt p53 sensitized cells to doxorubicin. A reduction of percentage of GFP positivity in surviving cells was observed.

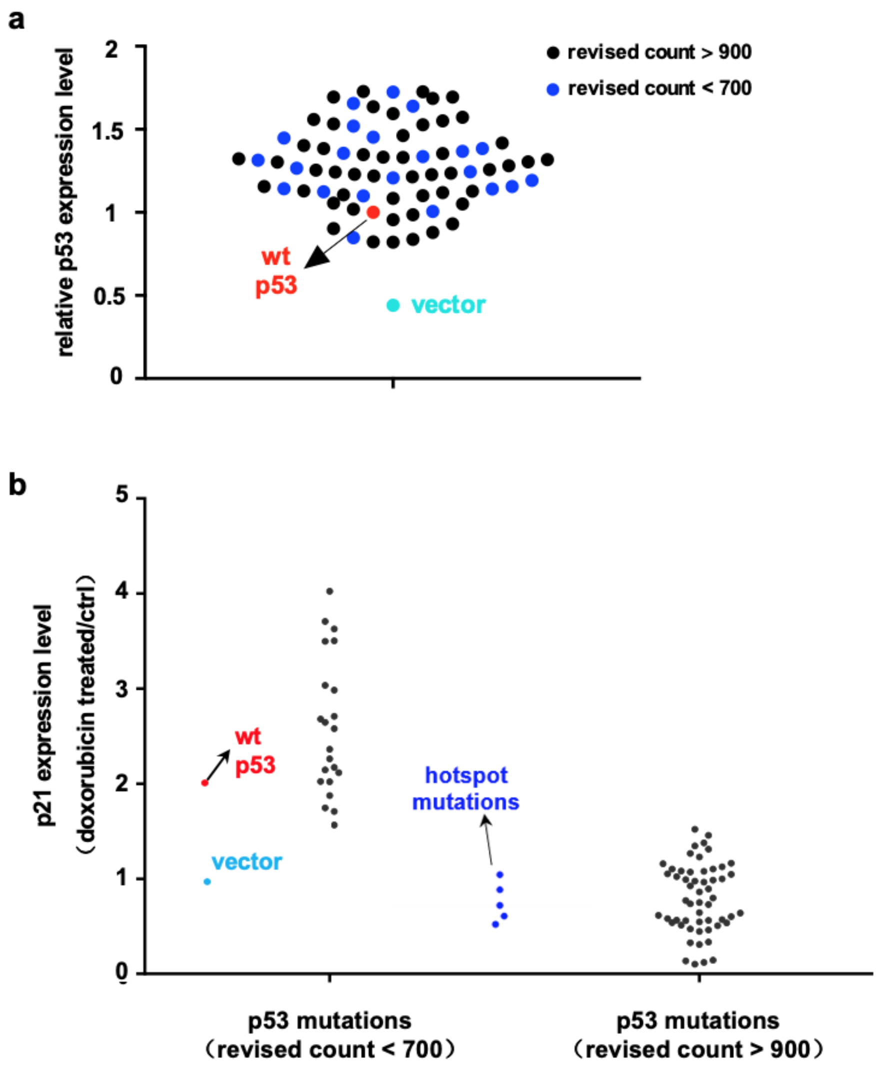

Figure S9. Transcriptional activities of different p53 mutants. (a) Expression levels of different p53 constructs were comparable in HCT116 p53-/- cells. (b) Retrovirus encoding mutant or wild type p53 was used to infect HCT116 p53-/- cells at 30-40% infection rate. A puromycin selection marker in the retrovirus were used to select infected cells. Such cells were then treated with doxorubicin for 24 hours, and the transcriptional activity of p53 mutants were analyzed by the mRNA level of p21.

a

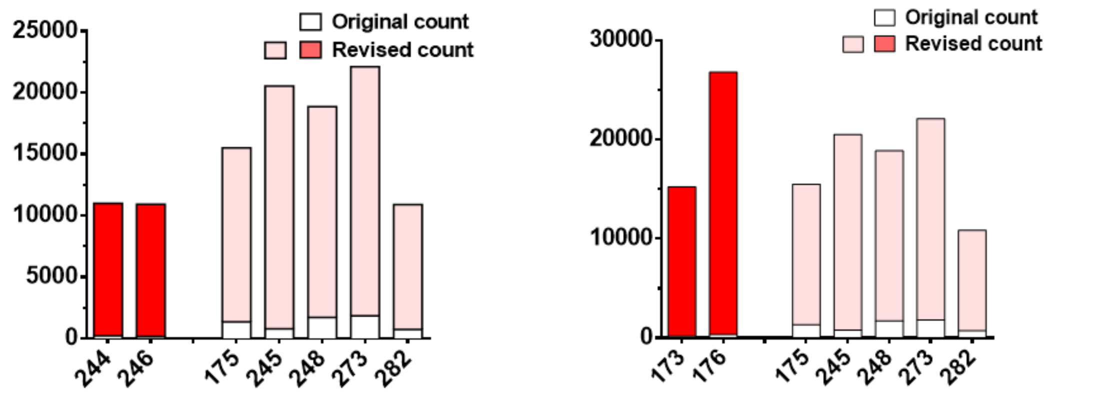

b

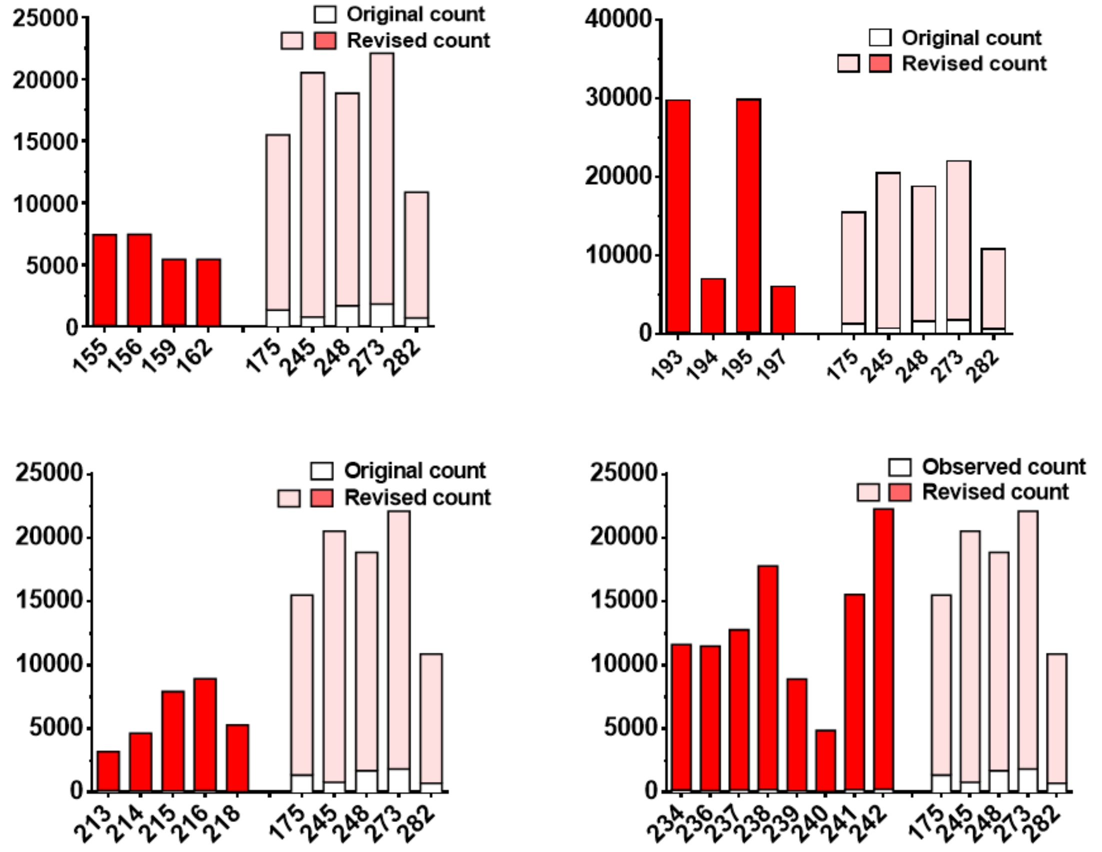

Figure S10. Additional functionally important amino acid residues and regions in p53. Original mutation counts of listed amino acid residues are indicated by white boxes, whereas revised counts are indicated by red or pink boxes. Hotspot mutation sites such as

R175 and G245 are included as controls. Shown here are stretches of functionally important amino acids in addition to those shown in Figure 4.

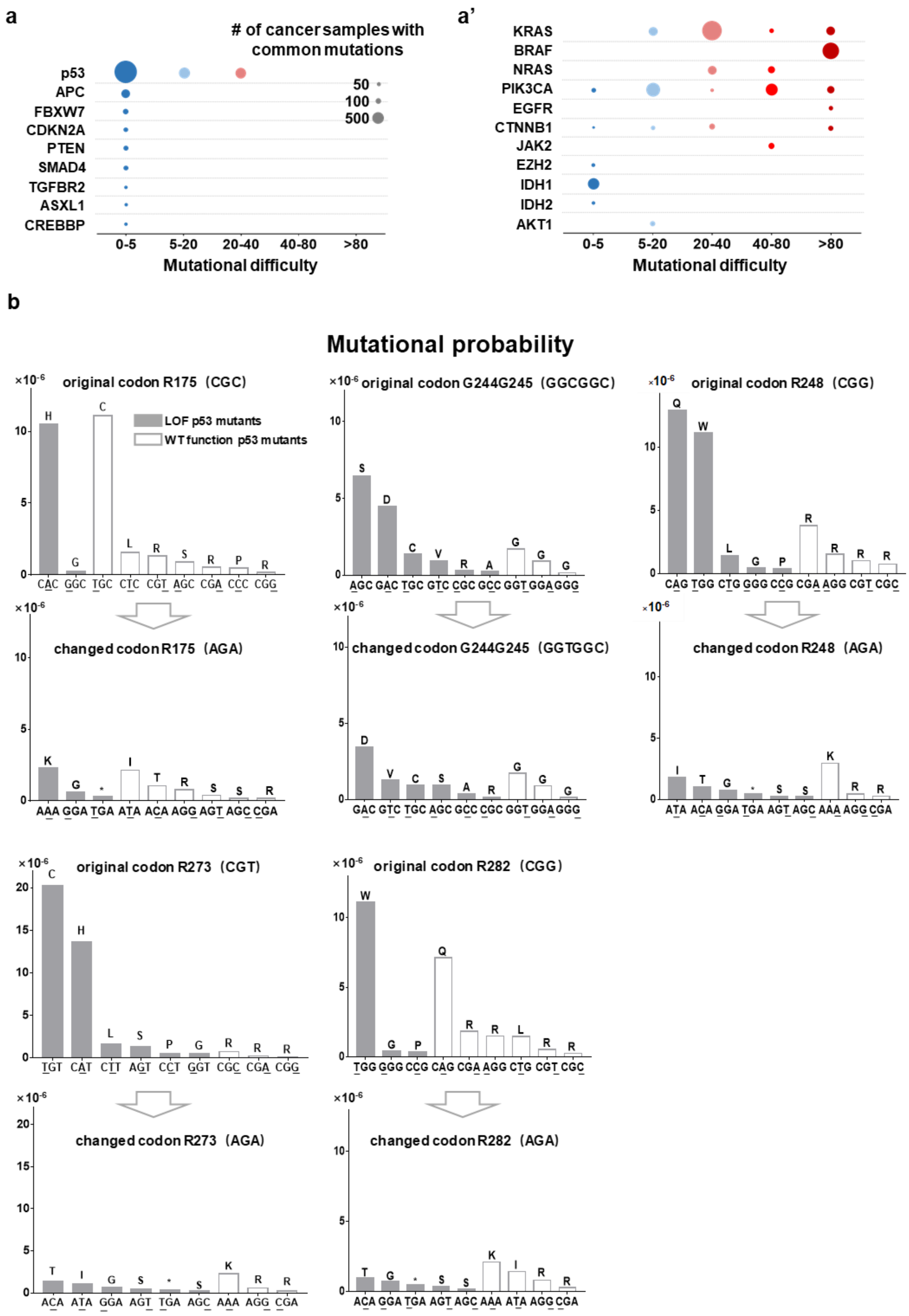
a
# of cancer samples with common mutations
50
100
500
p53
APC
FBXW7
CDKN2A
PTEN
SMAD4
TGFBR2
ASXL1
CREBBP
0-5
5-20
20-40
40-80
>80
Mutational difficulty
a'
KRAS
BRAF
NRAS
PIK3CA
EGFR
CTNNB1
JAK2
EZH2
IDH1
IDH2
AKT1
0-5
5-20
20-40
40-80
>80
Mutational difficulty
b
Mutational probability
original codon R175 (CGC)
LOF p53 mutants
WT function p53 mutants
changed codon R175 (AGA)
original codon G244G245 (GGCGGC)
changed codon G244G245 (GGTGGC)
original codon R248 (CGG)
changed codon R248 (AGA)
original codon R273 (CGT)
changed codon R273 (AGA)
original codon R282 (CGG)
changed codon R282 (AGA)

Figure S11. Potential implications for cancer prevention.

(a) and (a') Relative difficulties of common mutations on established tumor suppressors and oncogenes. Mutations on genes such as KRAS and p53 are collected from 26,154cancer genomes. Those mutations appearing in more than 30 cancer samples are plotted according to their relative mutational difficulties. For example, in (a) the blue dot adjacent to p53 represents how many cancer samples carry common p53 mutations whose relative mutational difficulties are between 0 and 5.

(b) Introduction of synonymous mutations at p53 hotspot sites can significantly reduce mutational probability. Loss of function mutations are shown in dark grey. Mutations with wild type p53 function are shown in white. As a result of such codon changes, novel mutations not in the COSMIC database may occur. The functional annotation of such p53 mutations (e.g. R175I) was made according to the PHANTM database. Of note, the high mutation rate on G245(GGC) is enabled by the preceding codon G244, which forms a nucleotide sequence (GCGGC) that is highly prone to G to A mutation, thereby causing the G245 (GGC) codon to be a hotspot mutation site. Change the G244 codon from GGC to GGT will decrease the mutation tendency of G245, without increasing mutation tendency of G244.

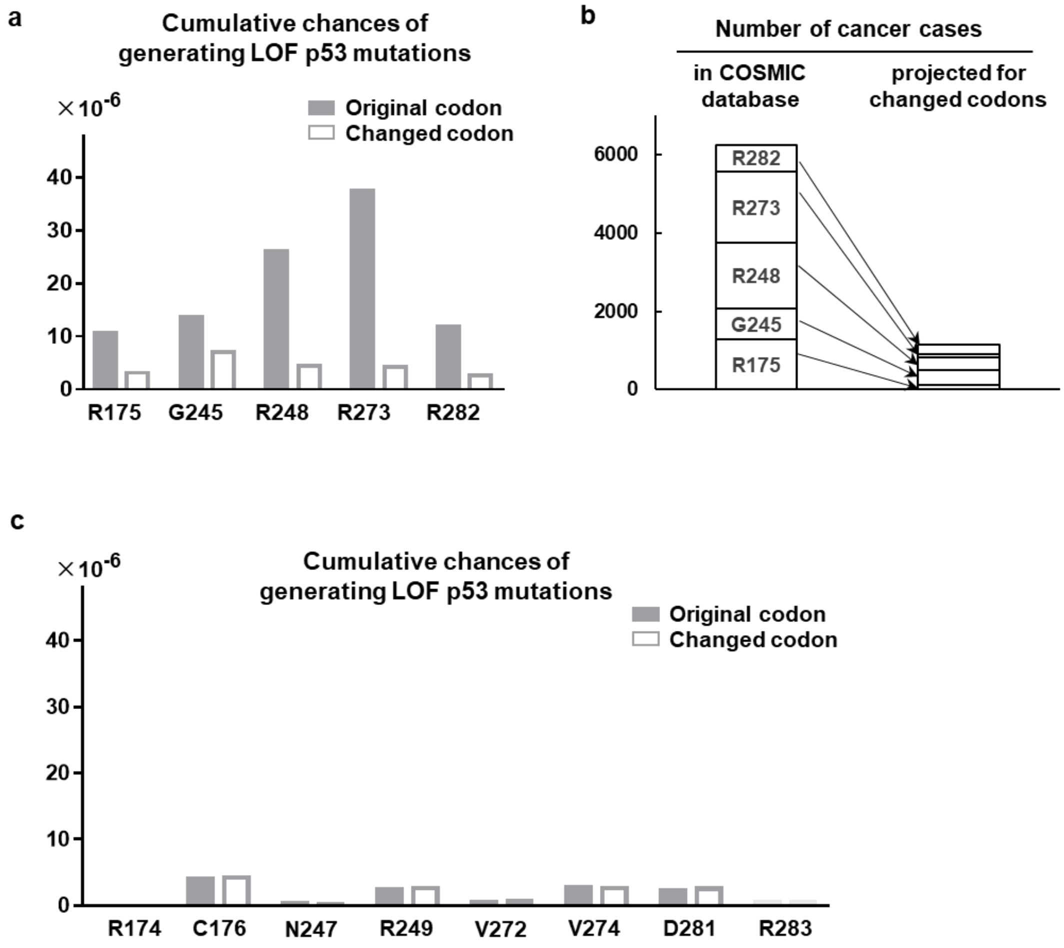

Figure S12. Potential impact of p53 codon changes on tumorigenesis.

(a) Cumulative chances of generating LOF p53 mutations on five hotspot mutation sites, based on original and changed codons.

(b) Number of cancer cases in COSMIC database involving these five p53 hotspot sites and the projected reduction of cancer cases if these sites are changed to hard-to-mutate codons.

(c) Condon changes on p53 hotspot sites do not significantly increase the chance of generating LOF p53 mutations at amino acid residues surrounding these hotspot sites.
